# Teachers’ Perspectives on Cyberbullying: A Cross-Cultural Study

**DOI:** 10.3390/ijerph19010257

**Published:** 2021-12-27

**Authors:** Almudena Castellanos, Beatriz Ortega-Ruipérez, David Aparisi

**Affiliations:** Faculty of Education, La Rioja International University, 26006 La Rioja, Spain; almudena.castellanos@unir.net (A.C.); beatriz.ortega.ruiperez@unir.net (B.O.-R.)

**Keywords:** cyberbullying, school bullying, perceptions, teaching skills, cross-cultural study

## Abstract

The aim of this work is to analyze the perceptions of Colombian, Spanish, and Ecuadorian teachers regarding cyberbullying from a cross-cultural perspective. A descriptive and analytical method was used with a quantitative approach and 240 teachers answered an ad hoc questionnaire. Most teachers in the three countries say that they do not know how to deal with this type of bullying and have not received training in this respect, with the percentages in the three countries being very similar. Spanish teachers have the highest percentage of lack of concern about cyberbullying and Colombian teachers are the ones who admit to having had the most cases of cyberbullying. In terms of reaction, the majority acted, but among those who did not, Ecuadorian teachers did not due to lack of knowledge. Forced by the pandemic to teach their classes online, teachers are increasingly concerned about cyberbullying. For the three countries, it is considered necessary to take measures in terms of legislating specific protocols to deal with cyberbullying at school and that the training plans for the degrees that give access to this profession include the competencies that allow teachers to develop appropriate strategies to respond to cyberbullying.

## 1. Introduction

Programs aimed at developing values, attitudes, and strategies, in both teachers and students, are necessary so that bullying situations do not occur; or, if they do, so that they last as short a time as possible and are not perpetuated because teachers have the tools to deal with these situations, reduce bullying, and improve the mental health problems of schoolchildren [1]. These are the conclusions of yet another study that points to the effectiveness of this type of intervention. Countries that are aware of this legislate on coexistence in their educational institutions. Despite this, UNESCO (United Nations Educational, Scientific and Cultural Organization) warned in its latest report on bullying of the global seriousness of the problem, with almost one in three students being affected [2]. The report also highlights that the percentage of bullying is higher in South America than in Europe.

According to students’ point of view in Spanish schools [3], and despite cyberbullying being one of the least observed types of bullying, the reality is that, in recent years, between 24% and 26% of school bullying cases are cyberbullying in the form of insults or threats, with the most used technological medium being the mobile phone by sending WhatsApp messages [4,5]. Recent research concludes that cyberbullying is higher in Latin American countries [6,7]. As for the role of teachers, according to [4], although the percentage of teachers who know about cases of cyberbullying, either because they have been informed or because they have noticed it, is very high, one third of teachers do not react to these cases because they play it down or do not believe the testimony. Over the years, teachers are taking more and more measures to solve cyberbullying, the main one being to talk to the bully, but also to inform the school management, the police, or seek the support of colleagues [8,9]. These results do not disagree with those of other studies conducted in Latin America, where victims are mostly of the opinion that teachers have done nothing to protect them [10]. From the teachers’ point of view, research concludes that the vast majority show concern about cyberbullying, although they do not consider it a problem in their educational center. Teachers need specific training, as many admit to not being able to recognize the problem and handle it [11,12,13], as well as not knowing about the existence of protocols for dealing with these situations [9]. Some point to the importance of the initial training of future teachers in these aspects [14]. On the other hand, there is research that has focused on asking victims about the actors that should help in the fight against online bullying. In the case of Europe, countries such as Spain point to the social media companies through which students are harassed, followed by the police. In Latin America, countries such as Colombia or Ecuador point to the government itself, in addition to these companies [6].

To help understand how Spanish, Colombian, and Ecuadorian teachers view cyberbullying, we first review the regulations that govern it in schools. In the case of Spain, article 124.1 of Organic Law 8/2013, of 9 December, for the Improvement of Educational Quality [15] clearly states that the educational project of the centers must include a coexistence plan. If we analyze the regulations concerning school coexistence in Colombia, as in Spain, it is the highest-ranking law, i.e., Law 115 of 8 February 1994, which establishes in its article 87 that educational establishments shall have a coexistence regulation or manual [16]. In the case of Ecuador, it is the Organic Law on Intercultural Education [17] which establishes that the school government of each educational establishment, as a participatory body of the educational community, will be responsible for drawing up the coexistence code. Each of the 17 Spanish Autonomous Regions has its own protocol for action against bullying; however, only four of them have ordered a specific protocol for cyberbullying [18]. The Colombian Ministry of Education has also not established a specific protocol for cyberbullying as such, although Decree 1965/2013 [19,20] defines what it is, classifying it as a Type II situation if it does not constitute a crime, and as a Type III situation if it does. Ecuador has not legislated a specific protocol for cases of cyberbullying and, like the other two countries, in 2017 the Ministry of Education published the document entitled Protocols for action in situations of violence committed in the education system [21].

## 2. Materials and Methods

### 2.1. Participants

To select the sample of individuals on whom the data were collected, a non-probabilistic sampling by accessibility [22] was used, with 240 teachers enrolled in a postgraduate course in educational technology and digital competencies taking part in the study. These teachers come from Spain (21.3%), Ecuador (33.3%), and Colombia (45.4%). In the case of Spanish teachers, they came from all the Autonomous Communities except Navarra, Asturias, and Castilla and León, with Cataluña being the most represented (21.6%), followed by Andalucía (15.7%). The Ecuadorian teachers come from 14 of Ecuador’s 24 provinces, the most frequent being Pichincha (36.2%)—where the capital is located—followed by Guayas (15%)—the other major urban center of the country. However, the sample also includes teachers from less populated areas, such as Loja (11.2%), and even from the Amazon, such as Orellana (1.2%). As for Colombian teachers, there are representatives from 17 of the 32 Departments into which Colombia is divided, with the highest percentage being Cundinamarca (33%)—which contains the country’s capital—followed by Antioquia (17.4%)—another of the most populated areas. As with teachers in Ecuador, the sample also includes teachers from less populated areas such as Risaralda (1.8%) or Quindío (1.8%). The 240 teachers in the sample work at different levels of education, from pre-school (0–6 years) to higher education (17 years and over), with those working in secondary education (12–16 years) being the most numerous (46.2%), followed by those working in primary education (6–12 years), who represent 27.1%. Less numerous are those working in higher education (17.1%) and in early childhood education (9.6%). Most teachers have been working for more than 4 years (67.1%) and 47.5% of the sample have more than 6 years of experience, but there are also teachers who have been working for between 1 and 3 years (20%) and even less than one year (12.9%).

### 2.2. Instruments

In accordance with the aim of the study, an ad hoc online questionnaire was designed to measure demographic and classification variables (country and autonomous community, province or department, educational level of specialization, and experience as a teacher) and variables related to knowledge and perspective on cyberbullying at school. The latter variables are collected in 14 dichotomous and polytomous items. The instrument is based on previous work on the subject [9,11]. To assess the degree of understanding of the questions and their suitability for the purpose of the work, we used the process of validation by judges [22], having selected 10 people who are experts in digital security in the field of education to individually evaluate each of the items. Their judgement of the instrument shows a level of agreement of 0.9 out of 1, which makes it valid for use [23].

### 2.3. Procedure

First, directors of different university master’s degrees belonging to faculties of education, whose students are active teachers, were contacted by e-mail. After an affirmative response to collaborate, they were contacted by telephone and provided with the necessary documentation to participate in the research. The questionnaire was administered in classrooms by staff from the centers themselves (generally a teacher from the master’s degree). It was emphasized that the questionnaire should be answered truthfully and without dwelling too long on each question. The time taken to complete the questionnaire ranged from 8 to 19 min. Collaboration was voluntary, anonymous, and disinterested. The study was carried out with the tacit consent of all participants and with the permission of the university centers.

### 2.4. Statistical Analyses

In accordance with the purpose of the study, to analyze in a comparative way the vision of Spanish, Colombian, and Ecuadorian teachers on school cyberbullying based on the regulations of their countries, we opted for a descriptive and analytical method with a quantitative approach. Descriptive statistics were used to analyze the demographic variables of the study participants. For the analysis of the relationship between the categorical variable “Country of residence” and the rest of the categorical variables, the cross-tabulation technique was used, in accordance with the objective of the study. The Chi-square test was applied to study the relationship between the categorical variables and the standardized residuals were analyzed to ascertain the direction in which the relationship between the variables studied occurred. The significance level was 0.05. All analyses were carried out using SPSS version 26.0 statistical software (by IBM, Armonk, NY, USA).

## 3. Results

Table 1 shows that the majority (77.1%) of the teachers consulted can confirm the existence of a coexistence manual in their institution. A percentage of 16.2% did not know whether such a document existed in their institution and, according to some teachers (6.7%), their institution did not even have any document regulating coexistence. In this regard, it is interesting to note that Spanish teachers account for the highest percentage (33.3%) who do not know whether a coexistence plan exists in their educational center, while Ecuadorian teachers are the ones who mainly (10%) answered that their centers do not have a coexistence plan.

Table 2 reveals that 42.9% of teachers have not received training on coexistence in the center where they work, and in the case of those resident in Spain, it is more than half (54.9%).

Table 3 refers to the protocol for action against bullying. 47.9% of those surveyed stated that their school has such a document, with teachers residing in Colombia and Ecuador being the ones who most acknowledged that they do not have it.

In Table 4, concerning specific protocols for cases of cyberbullying, the majority (50.8%) of teachers recognize that there is no such instrument in their educational center—61.3% in the case of Colombian teachers, 53.7% in the case of Ecuadorian teachers, and 23.5% of Spanish teachers—or they are unaware of it (36.2%). Mainly Spanish teachers (62.8%) are unaware of such an instrument in their educational center.

Table 5 shows that 74.6% of teachers do not know how to act in the event of cyberbullying. There is not much difference between the three countries in terms of the percentage who say they do not know how to act—78.4% of Spaniards, 74.3% of Colombians, and 72.5% of Ecuadorians.

According to the percentages in Table 6, 90.8% of the subjects surveyed have not received training on cyberbullying in the studies that allowed them to enter the teaching profession. Of those who say they have received training, the highest percentage (12.5%) corresponds to teachers from Ecuador.

Similarly, the majority (78.7%) of teachers say that they have not received training on cyberbullying in the center where they work (Table 7). Professionals from Colombia are the ones who represent the highest percentage (24.8%) of those who claim to have received training in cyberbullying (Table 7).

As Table 8 shows, 97.1% of the teachers surveyed are concerned about cyberbullying, with Colombian teachers having the lowest percentage of concern (95.4%). 95.4% consider it to be a current problem in schools and, in this case, Spanish teachers have the lowest percentage of concern (90.2%), as shown in Table 9.

Additionally, 23.8% of the sample (Table 10) have experienced a case of cyberbullying as a teacher, with the highest percentage in Colombia (33%), followed by Spain (21.6%).

Regarding whether teachers act in cases of cyberbullying, Table 11 shows that 89.5% of teachers who had been presented with a case acted and, of the three countries, Ecuador has the highest percentage of negative responses (20%). Regarding the reasons for not acting, of the responses collected, the cause with the highest percentage (50%) is “Lack of knowledge” when it comes to acting, as shown in Table 12.

Of the 57 teachers who stated that they had taken measures when they found out about a case of cyberbullying, 50 responded to the type of measure they took (Table 13), with the highest percentage being “notify the specific coexistence team” (34%), “talk to those involved” (28%), and “contact other figures or organizations” (26%). It is Colombian and Ecuadorian teachers—45.4% and 33.3%, respectively—who prefer the measure “notify the specific coexistence team”, while Spaniards are more in favor of “dialogue with those involved” (36.3%).

Regarding the Digital Plan for the school, Spanish teachers are the ones with the highest percentage (33.3%) who state that their institution has such a document (Table 14).

Finally, the relationships between the qualitative variables were analyzed and the following results were obtained. The Chi-square statistic indicated (Table 15) statistically significant relationships between the variables “Do you know how to act in the event of cyberbullying” and “Educational level” (*p* < 0.05), showing that secondary school teachers who selected that they do know how to act in the event of cyberbullying have a significantly higher frequency than expected. Between the variables “Have you received training in coexistence at the educational centre where you work” and “Experience as a teacher” (*p* < 0.05), there is a significantly higher frequency of teachers who have received training in coexistence at the educational centre where they work (*p* < 0.05). Between the variables “Have you received training in coexistence at the educational centre where you work” and “Experience as a teacher” (*p* < 0.05), there is a significantly higher frequency of teachers who have received training at their centre and have more years of experience. Finally, the variables “Does your centre have a coexistence plan” and “Have you taken any measures in the event of cyberbullying” (*p* < 0.05) have a significantly higher frequency than expected (*p* < 0.05), showing that teachers who claim not to have a coexistence plan answered that they did not take measures in cases of cyberbullying with a significantly higher frequency than expected.

## 4. Discussion

The aim of this study was to analyze the perceptions of Spanish, Colombian, and Ecuadorian teachers regarding cyberbullying from a comparative perspective. The efforts of the three countries to regulate coexistence in schools and to involve teachers in contributing to it seem to be reflected in the number of teachers who are aware that their school has a coexistence manual. Despite this, there are still cases where there is no such manual, even though it is compulsory and public in all three countries. It is also worth noting the percentage who say that they have not received training on coexistence, even though legislation in all three countries encourages these documents regulating coexistence to be sufficiently disseminated. In the case of protocols for dealing with bullying, this document is less well known by teachers in the three countries, which may be related to the fact that fewer regulations have been published on the subject. About specific protocols for cyberbullying cases, most teachers confirm their non-existence, a response that is consistent with the scarcity of approved regulations and with the limited competence to act when faced with a case of cyberbullying at school, which other studies also agree with [9,11,12,13].

For years, teachers have been calling for training in this type of bullying [11] since, as the data from this study show, most of the teachers consulted state that they have not received training in cyberbullying during the studies that allowed them to enter the teaching profession, as confirmed by other research [14], nor have they received training from the institution in which they work. In this same sense, practically all respondents state that cyberbullying is an issue that concerns them as teachers and that they also consider it a current problem in schools forced by the Covid-19 pandemic to teach classes online. This is a change from other similar studies where teachers express their concern, but do not consider it a problem in their school [11]. It is interesting to note the significant relationship between teachers who know how to act in cases of cyberbullying and the educational level at which they teach (12–16 years), which is closely related to the average age of the victims [4]. The observed cases of cyberbullying are higher in Colombia, which could be related to the tendency for Latin American countries to have a higher number of cases [6,7].

As for the reaction of teachers to these cases, most teachers who have been informed of cyberbullying have taken some action, even though, as mentioned above, they claim not to know how to deal with this problem. Among the teachers who did not act, the reasons given point to a lack of knowledge when it comes to dealing with the problem. In other previous studies, the percentage of teachers who have acted in cases of cyberbullying is lower and the reasons for not doing so are more varied, such as downplaying the importance, not believing the testimony, etc. [4]. As for the group of teachers who recognize that they have acted in cases of cyberbullying, the strategies they adopt are mostly communication strategies, and these data are like those reflected in other studies [8]. If we make a comparative analysis, Colombian teachers are the most willing to explain the measures they have taken in cases of cyberbullying, followed by Spanish teachers, the former being the ones with the most consistent response, where the tendency to follow the protocol and report to the school’s coexistence committee can be seen.

At a practical level, these results can guide the development of intervention programs [24] aimed at all teachers to promote awareness of the issue of cyberbullying and its repercussions, both on a personal [25,26] and academic [27] level, as well as to work with families and parenting styles as a protective factor [28].

## 5. Conclusions

In conclusion, for all three countries, it is considered necessary to take measures in two areas: teacher training and the design of specific protocols for cyberbullying. Regarding equipping teachers with skills, it is essential that the training plans for the degrees that give access to this profession consider the need expressed in this study and include skills to develop appropriate strategies for responding to cyberbullying, identifying, and responding to online behaviors and safety problems, and ensuring the correctness of behaviors and actions when interacting with other people online, following the recommendations of [29]. In terms of regulating protocols for cyberbullying, it is recommended that countries legislate a model of action for the educational community [30] in the same way that coexistence manuals or protocols for traditional bullying are already in place.

## Figures and Tables

**Table 1:** Cross categories of the variable “Does your educational center have a Coexistence Plan” and the variable “Country of Residence”.

Does Your School Have a Coexistence Code/Manual/Plan/Project?			Country of Residence			Total
			Colombia	Ecuador	Spain	
Answers	I don’t know	n	12	10	17	39
		%	11.0%	12.5%	33.3%	16.2%
	No	n	6	8	2	16
		%	5.5%	10.0%	4.0%	6.7%
	Yes	n	91	62	32	185
		%	83.5%	77.5%	62.7%	77.1%
Total		N	109	80	51	240
			100%	100%	100%	100%

**Table 2:** Cross-categorization of the variable “Have you received training in Coexistence in the educational center where you work?” and the variable “Country of Residence”.

Have You Received Training on Coexistence in the Educational Center Where You Work or Have Worked?			Country of Residence			Total
			Colombia	Ecuador	Spain	
Answers	No	n	41	34	28	103
		%	37.6%	42.5%	54.9%	42.9%
	Yes	n	68	46	23	137
		%	62.4%	57.5%	45.1%	57.1%
Total		N	109	80	51	240
		%	100%	100%	100%	100%

**Table 3:** Cross-categorization of the variable “Does your school have a bullying protocol” and the variable “Country of residence”.

Does Your School Have a Protocol for Dealing with Bullying?			Country of Residence			Total
			Colombia	Ecuador	Spain	
Answers	I don’t know	n	23	14	24	61
		%	21.1%	17.5%	47.1%	25.4%
	No	n	34	25	5	64
		%	31.2%	31.2%	9.8%	26.7%
	Yes	n	52	41	22	115
		%	47.7%	51.3%	43.1%	47.9%
Total		N	109	80	51	240
		%	100%	100%	100%	100%

**Table 4:** Cross-categorization of the variable “Does your school have a specific protocol for cyberbullying cases” and the variable “Country of residence”.

Does Your School Have a Specific Protocol for Dealing with Cyberbullying?			Country of Residence			Total
			Colombia	Ecuador	Spain	
Answers	I don’t know	n	27	28	32	87
		%	24.8%	35.0%	62.8%	36.2%
	No	n	67	43	12	122
		%	61.3%	53.7%	23.5%	50.8%
	Yes	n	15	9	7	31
		%	13.8%	11.3%	13.7%	13.0%
Total		N	109	80	51	240
		%	100%	100%	100%	100%

**Table 5:** Cross-categorization of the variable “Do you know how to deal with cyberbullying?” and the variable “Country of residence”.

Do You as a Teacher Know What to Do in Case of Cyberbullying?			Country of Residence			Total
			Colombia	Ecuador	Spain	
Answers	No	n	81	58	40	179
		%	74.3%	72.5%	78.4%	74.6%
	Yes	n	28	22	11	61
		%	25.7%	27.5%	21.6%	25.4%
Total		N	109	80	51	240
		%	100%	100%	100%	100%

**Table 6:** Cross-categorization of the variable “In the studies that allowed you to enter the teaching profession, did you receive training on cyberbullying” and the variable “Country of residence”.

Did You Receive Training on Cyberbullying in Your Studies Leading to Your Entry to the Teaching Profession?			Country of Residence			Total
			Colombia	Ecuador	Spain	
Answers	No	n	101	70	47	218
		%	92.7%	87.5%	92.2%	90.8%
	Yes	n	8	10	4	22
		%	7.3%	12.5%	7.8%	9.2%
Total		N	109	80	51	240
		%	100%	100%	100%	100%

**Table 7:** Cross-categorization of the variable “Have you received training on cyberbullying in the center where you work” and the variable “Country of residence”.

Have You Received Training on Cyberbullying in the School Where You Work or Have Worked?			Country of Residence			Total
			Colombia	Ecuador	Spain	
Answers	No	n	82	65	42	189
		%	75.2%	81.3%	82.4%	78.7%
	Yes	n	27	15	9	51
		%	24.8%	18.7%	17.6%	21.3%
Total		N	109	80	51	240
		%	100%	100%	100%	100%

**Table 8:** Cross-categorization of the variable “Are you concerned about cyberbullying as a teacher?” and the variable “Country of Residence”.

Are You Concerned about Cyberbullying as a Teacher?			Country of Residence			Total
			Colombia	Ecuador	Spain	
Answers	No	n	5	1	1	7
		%	4.6%	1.2%	2.0%	2.9%
	Yes	n	104	79	50	233
		%	95.4%	98.8%	98%	97.1%
Total		N	109	80	51	240
		%	100%	100%	100%	100%

**Table 9:** Cross-categorization of the variable “Do you consider cyberbullying a current problem in schools?” and the variable “Country of Residence”.

Do You Consider Cyberbullying to Be a Current Problem in Schools?			Country of Residence			Total
			Colombia	Ecuador	Spain	
Answers	No	n	4	2	5	11
		%	3.7%	2.5%	9.8%	4.6%
	Yes	n	105	78	46	229
		%	96.3%	97.5%	90.2%	95.4%
Total		N	109	80	51	240
		%	100%	100%	100%	100%

**Table 10:** Cross-categorization of the variable “Have you observed cyberbullying as a teacher?” and the variable “Country of Residence”.

Have Your Students Reported Cyberbullying to You or Have You Observed Cyberbullying as a Teacher?			Country of Residence			Total
			Colombia	Ecuador	Spain	
Answers	No	n	73	70	40	183
		%	67.0%	87.5%	78.4%	76.2%
	Yes	n	36	10	11	57
		%	33%	12.5%	21.6%	23.8%
Total		N	109	80	51	240
		%	100%	100%	100%	100%

**Table 11:** Cross-categorization of the variable “Have you taken any action in case of cyberbullying?” and the variable “Country of residence”.

Have You Taken Any Action When You Have Been Informed of or Become Aware of a Case of Cyberbullying?			Country of Residence			Total
			Colombia	Ecuador	Spain	
Answers	No	n	3	2	1	6
		%	8.3%	20.0%	9.1%	10.5%
	Yes	n	33	8	10	51
		%	91.7%	80%	90.1%	89.5%
Total		N	36	10	11	57
		%	100%	100%	100%	100%

**Table 12:** Cross-categorisation of the variable “Main reason for not taking action when aware of cyberbullying” and the variable “Country of residence”.

Main Reason for Not Acting When Aware of a Case of Cyberbullying			Country			Total
			Colombia	Ecuador	Spain	
Answers	Lack of awareness	n	1	1	0	2
		%	33.3%	100%	0%	50%
	Downplaying the case	n	0	0	0	0
		%	0%	0%	0%	0%
	Distrust testimony	n	1	0	0	1
		%	33.3%	0%	0%	25%
	Others	n	1	0	0	1
		%	33.3%	0%	0%	25%
Total		N	3	1	0	4
		%	100%	100%	0%	100%

**Table 13:** Cross-categorization of the variable “Actions you have taken in case of observing cyberbullying” and the variable “Country of residence”.

Actions You Have Taken If You Know of or Observe Cyberbullying			Country of Residence			Total
			Colombia	Ecuador	Spain	
Answers	Notify the person responsible for coexistence	n	15	2	0	17
		%	45.4%	33.3%	0%	34%
	Dialogue with those involved	n	9	1	4	14
		%	27.3%	16.7%	36.3%	28%
	Dialogue with the family	n	2	0	3	5
		%	6.1%	0%	27.3%	10%
	Punitive Measures	n	0	0	1	1
		%	0%	0%	9.1%	2%
	Contact with other figures or organizations (psychologist, director, social worker...)	n	7	3	3	13
		%	21.2%	50%	27.3%	26%
Total		N	33	6	11	50
		%	100%	100%	100%	100%

**Table 14:** Cross-categorization of the variable “Do you know if your educational institution has a Digital School Plan?” and the variable “Country of Residence”.

Do You Know If Your Educational Institution Has a Digital School Plan?			Country of Residence			Total
			Colombia	Ecuador	Spain	
Answers	I don’t know	n	36	37	26	99
		%	33%	46.2%	51%	41.3%
	No Digital Plan	n	62	40	8	110
		%	56.9%	50%	15.7%	45.8%
	It does have a Digital Plan	n	11	3	17	31
		%	10.1%	3.8%	33.3%	12.9%
Total		N	109	80	51	240
		%	100%	100%	100%	100%

**Table 15:** Results derived from Pearson’s Chi-square test for independence.

Pearson’s *Chi-square* Test	Value	*df*	Signif. (bil.)
Do you know how to act in case of cyberbullying?/Education level	9.4	3	0.02
Have you received training in Coexistence in the educational center where you work?/Teaching experience	9.7	3	0.02
Does your educational center have a Coexistence Plan?/Have you taken any action when you have become aware of a case of cyberbullying?	9.56	2	0.00

Note. Signif. (bil.) = Significance (bilateral).

## Data Availability

The data presented in this study are available on request from the corresponding author.
